# Nonlinear internal wave spirals in the northern East China Sea

**DOI:** 10.1038/s41598-018-21461-3

**Published:** 2018-02-22

**Authors:** SungHyun Nam, Duk-jin Kim, Seung-Woo Lee, Bong Guk Kim, Ki-mook Kang, Yang-Ki Cho

**Affiliations:** 1 School of Earth and Environmental Sciences, Seoul National University, Seoul, 08826 Republic of Korea; 2 Research Institute of Oceanography, Seoul National University, Seoul, 08826 Republic of Korea

## Abstract

Oceanic internal waves are known to be important to the understanding of underwater acoustics, marine biogeochemistry, submarine navigation and engineering, and the Earth’s climate. In spite of the importance and increased knowledge of their ubiquity, the wave generation is still poorly understood in most parts of the world’s oceans. Here, we use satellite synthetic aperture radar images, *in-situ* observations, and numerical models to (1) show that wave energy (having relatively high amplitude) radiates from a shallow sill in the East China Sea in all directions, but with a significant time lag dependent on background conditions, (2) reveal that wave fronts are locally formed with often favorable conditions for re-initiation, and (3) demonstrate the resulting variety of wave patterns. These findings would be the case for any broad shelf having shallow sills with time-varying conditions, and therefore have significant implications on the redistribution of energy and materials in the global as well as regional ocean.

## Introduction

Oceanic nonlinear internal waves (NIWs) are known to play an important role in transporting and redistributing heat, mass, energy, and materials, and to be important to the understanding of underwater acoustics^1^, submarine navigation and offshore engineering^2^, marine biogeochemistry^3,4^, and the Earth’s climate^5–7^. As ubiquitously found in the ocean and primarily generated by the wind and the tides, oceanic nonlinear internal waves (NIWs) of various forms (e.g., arc-like or parabolic forms originating from a single point or a line) have been examined in the past decades using both theories^8–11^ and observations^12–15^. They have been understood primarily in the contexts of the Korteweg deVries equation where the nonlinear and dispersive effects are comparable to form a solitary shape^8^, and the Taylor-Goldstein equations, where the propagation speeds (eigenvalues) and vertical structures (eigenvectors) are obtained for long wavelength, small amplitude, and hydrostatic perturbations^16^. More recently, a variable-coefficient Ostrovsky equation was derived to better understand the deformation and transformation as well as propagation of NIWs by taking the combined effects of the Earth’s rotation and bathymetric changes into account^11,17^. Yet, our understanding of their mechanisms of generation, propagation, deformation, and transformation relies on a few limited observations^13–15^, mostly due to the challenges involved in the collection of *in situ* data with a necessary resolution and the limitation of active synthetic aperture radar (SAR) sampling from polar-orbit satellites.

**Figure 1:**
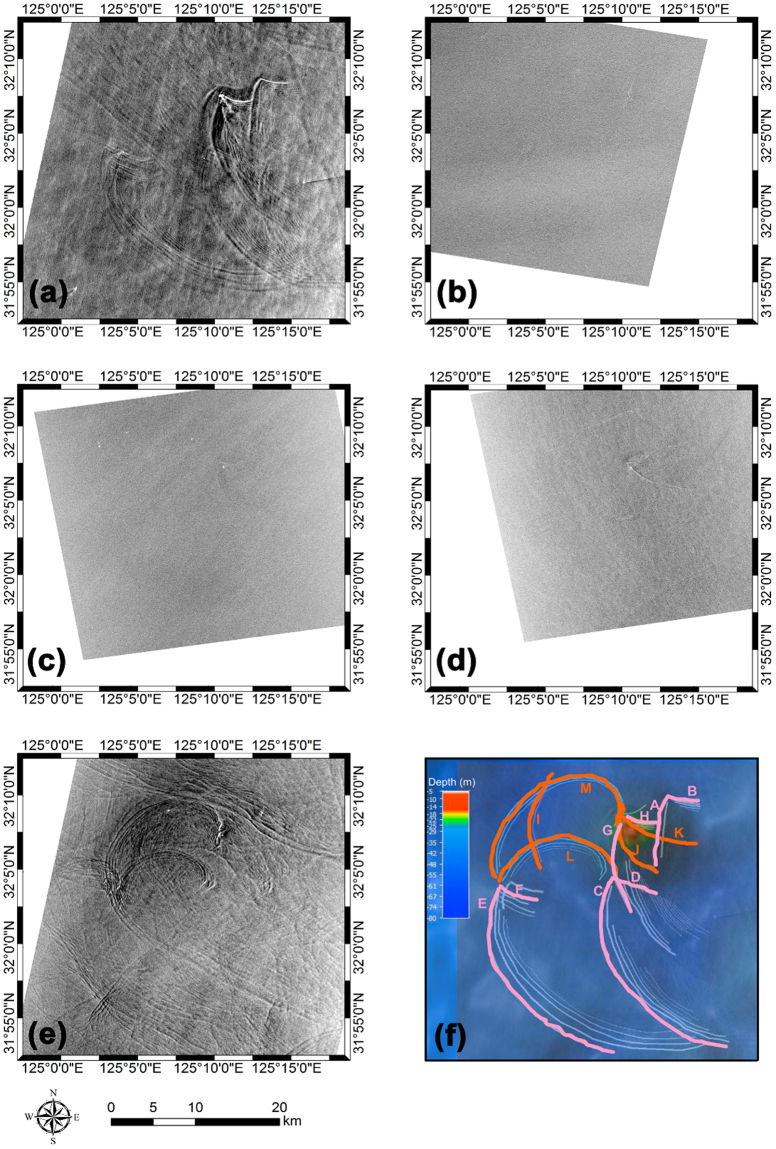
Synthetic aperture radar (SAR) images in the vicinity of a small topographic sill (submerged rock known as IEODO, 32°N 7.38′, 125°E 10.95′) in the northern East China Sea. SAR images taken on (**a**) 21:45:30 UTC September 10, 2014, (**b**) 09:43:54 UTC May 28, 2015, (**c**) 21:38:07 UTC June 3, 2015, (**d**) 21:31:17 UTC June 5, 2015, (**e**) 21:45:31 UTC June 12, 2015, and (**f**) bottom topography around the sill (color scale in the top-left), superimposed with a surface manifestation of wave fronts where the increased sea surface roughness is found in association with convergences of near-surface currents induced by internal waves (from ‘A’ to ‘H’ in 2014; pink, from ‘I’ to ‘M’ in 2015; orange) with an indication of directional measure of distance (distance from a moving local origin as a function of an angle difference *θ* in degree rotated counterclockwise from the east).

Limited to several snapshots of satellite imagery, such as SAR images^1^, and fewer *in-situ* measurements reported to date^18–23^, our understanding of the generation of East China Sea (ECS) NIWs remains in a primitive state, particularly in contrast to those in the South China Sea (SCS)^15^. It is generally believed that the NIWs are formed mainly by strong tidal forced interactions with bathymetric features, such as topographic sill, seamount, island, and shelf break. Among many others, there is a small sill with a depth as shallow as 5 m, where the Republic of Korea’s IEODO ocean research station (IORS) is located. Based on theories, seasonal variations of propagating speed and characteristic wavelength of mode-1 NIWs were estimated using historical hydrographic data collected in the northern ECS over a few decades^24^. Since the wave speed is the same order of magnitude (O (10^−1^–10^0^) m s^−1^) as that of local tidal flow (speed of predominantly semidiurnal M_2_ tidal currents) over the sill in stratified seasons, there is a high possibility that the NIWs generated at semidiurnal frequency propagate forward and backward from the sill depending on these speeds. From the variety of forms of ECS NIW fronts seen in the SAR images and analysis of *in-situ* data, multiple directions of propagation from multiple origins were suggested or more commonly speculated^19,20,24^. However, patterns of NIW fronts in the vicinity of IORS have not been addressed from observations. In particular, any mechanism for formation of the various (often unexpected) patterns of NIW fronts observed around the sill or any underwater conditions stressing NIW generation have not been unraveled until the present study. Thus, here we present the results of an analysis on the NIW features in SAR images recently taken around the IORS (Fig. 1, Table 1) in accordance with the analysis of modeled conditions of regional circulation.

**Table 1:** Times of SAR observations, flow regimes, characteristics and names of surface frontal feature of waves, and estimated times of wave generation.

SAR image	Time (SAR image taken)	Flow regime and characteristics of internal wave features	Feature name /Time of wave generation
#1	21:45:30 September 10, 2014	Wedge patterns consisting of long and short arc-like curvatures extending from the sill	‘B’/22:05 Sep 8, 2014 ‘A’/23:55 Sep 8, 2014 ‘D’/11:40 Sep 9, 2014 ‘C’/11:50 Sep 9, 2014 ‘F’/22:20 Sep 9, 2014 ‘E’/00:15 Sep 10, 2014 ‘H’/10:10 Sep 10, 2014 ‘G’/12:40 Sep 10, 2014
#2	09:43:54 May 28, 2015	Propagation dominated, waves generated from a remote area passing by the sill	No local generation
#3	21:38:07 June 3, 2015	Short arc-like curvatures starting from the sill	‘J’/19:55 Jun 3, 2015
#4	21:31:17 June 5, 2015	Short arc-like curvatures starting from the sill	‘K’/19:44 Jun 3, 2015
#5	21:45:31 June 12, 2015	Long and spiral patterns starting from the sill	‘L’/06:05 Jun 12, 2015, ‘M’/16:50 Jun 12, 2015

## Results and Discussion

### Regional ocean circulation model

**Figure 2:**
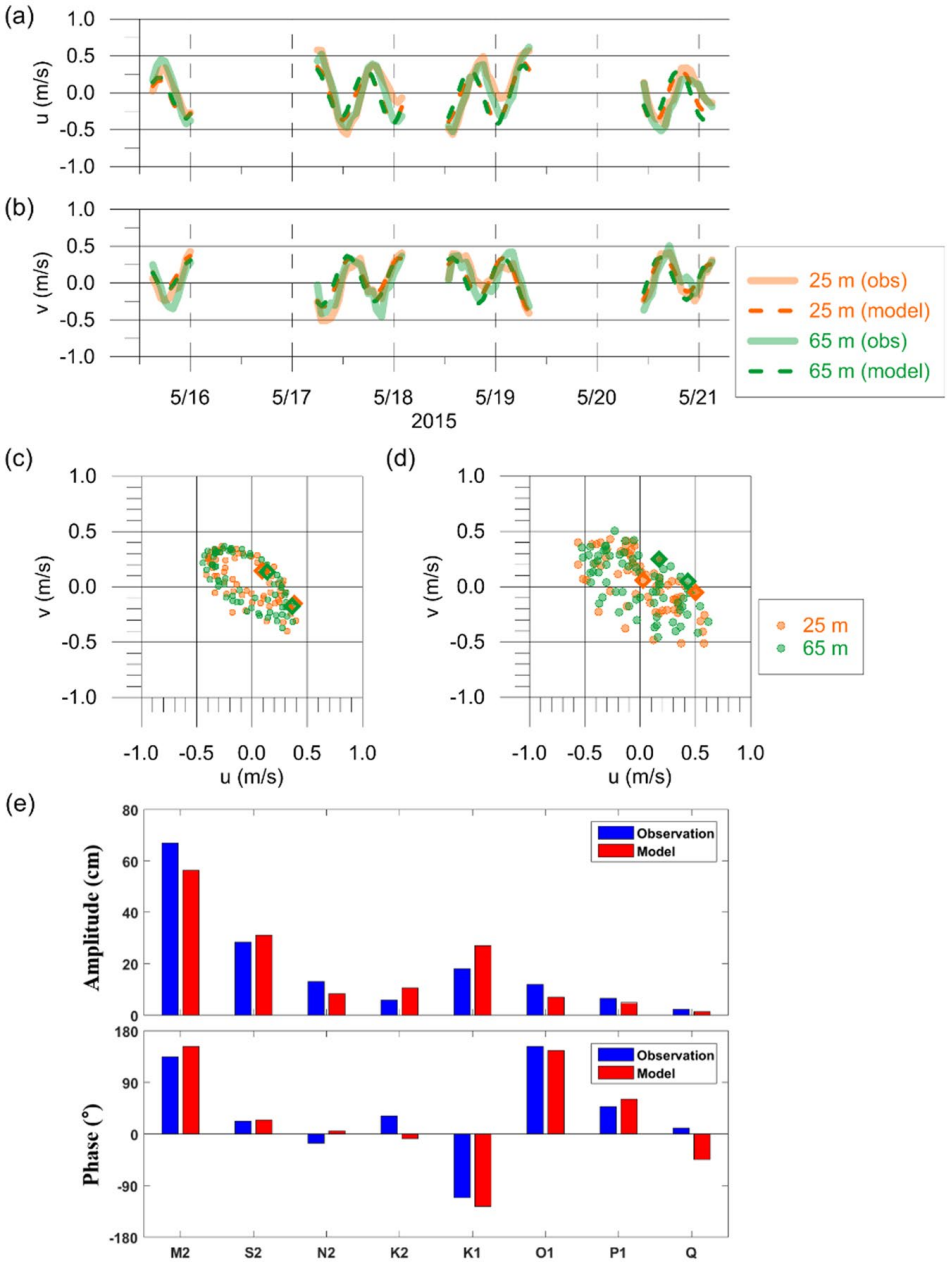
Time series of (**a**) zonal and (**b**) meridional components and (**c**,**d**) hodographs of absolute (mostly tidal) horizontal current vectors at the upper (25 m, orange) and lower (65 m, green) depths in the SAVEX-15 area (water depth: 100 m), ~50 km east of the IORS in May 2015. Time series of (**a**) zonal and (**b**) meridional components and (**c**,**d**) hodographs of absolute (tidal plus subtidal) horizontal current vectors at the upper (25 m, orange) and lower (65 m, green) depths in the SAVEX-15 area (water depth: 100 m), ~50 km east of the IORS in May 2015. In (**a**) and (**b**), each component observed during the SAVEX-15 cruise (thick solid line) and reproduced by the ROMS (dashed line) are superimposed. The reproduced and observed horizontal current vectors are shown by circles with an interval of one hour in (**c**) and (**d**), respectively. Open diamond symbols denote a time difference of 48.00 hours to compare the relative phase of tidal currents over the period shown in (**a**) and (**b**). The model data during the observational gap period caused by acoustic experiments (when the ADCP turned off) were not used here. In (**e**), shown are the tidal amplitudes and phases of for 8 major tidal constituents reproduced by the ROMS (red) and observed at the IORS (blue) for the period from April to June 2015.

Tidal oscillations (tidal elevation and tidal currents) in addition to regional circulation and their seasonal changes were reasonably simulated using a Regional Ocean Modeling System (ROMS)^25^, which provides a realistic background condition for NIWs – vertical density stratification and horizontal currents (tidal plus subtidal) at multiple layers of the IORS (validated with *in-situ* observations; see Methods section for details; e.g., Fig. 2). Two seasons – September 2014 and May to June 2015 - were selected based on when the SAR images were taken around the IORS. Specifications of SAR images including sea surface wind and wave conditions are provided in Table 2 (see Methods section for details). Modeled sea surface temperature and volume transport across the straits in the region had consistent features with observations of sea surface temperature^25^ and volume transport^25^.

**Table 2:** Specification of SAR images with sea surface wind and wave conditions.

Figure 1.	(a)	(b)	(c)	(d)	(e)
Satellite name	TanDEM-X	KOMPSAT-5	KOMPSAT-5	KOMPSAT-5	TanDEM-X
Acquisition time	21:45:30 UTC Sep. 10, 2014	09:43:54 UTC May 28, 2015	21:38:07 UTC June 3, 2015	21:31:17 UTC June 5, 2015	21:45:31 UTC June 12, 2015
Band	X-band	X-band	X-band	X-band	X-band
Orbit direction	Descending	Descending	Ascending	Ascending	Descending
Polarization	VV	VV	VV	VV	VV
Incidence angle (˚)	31.7–34.7	22.3–25.0	47.1–48.8	36.5–38.6	32.0–34.9
Wind speed (m s^−1^)	7.3	7.4	7.5	5.2	7.5
Wind direction (˚)	38	103	41	178	212
Significant wave height (m)	1.2	1.6	1.7	0.8	0.8

### Flow regime and directional wave evolution

The Froude number (*Fr*), used as the key parameter analyzed here to determine the timing of NIW generation, is given as the ratio between advective flow speed and free wave speed. To consider strong time-varying background horizontal flow (primarily tidal current here) as well as density stratification, the composite Froude number for water column can be defined as below^26^:

1 $${G}^{2}=\sum _{i=1}^{N}{{F}_{i}}^{2}$$

where *N* defines the number of layers, and

2 $${{F}_{i}}^{2}=\frac{{{U}_{i}}^{2}}{{({c}_{i}-{U}_{i})}^{2}}$$

where ${U}_{i}=\sqrt{{{u}_{i}}^{2}+{{v}_{i}}^{2}}$ and ${c}_{i}=\sqrt{g^{\prime} {h}_{i}}+{U}_{i}$ are the advective flow speed and apparent free wave speed incorporating an effective Doppler shift due to the advective flow at each layer, respectively. $g^{\prime} =g{\rm{\Delta }}{\rho }_{i}/{\rho }_{i}$ is reduced gravity determined by the density ratios between layers, ${\rm{\Delta }}{\rho }_{i}=({\rho }_{i+1}-{\rho }_{i-1})/2$ for *i* ≠ 1 and *i* ≠ *N*, ${\rm{\Delta }}{\rho }_{1}={\rho }_{2}-{\rho }_{1}$, ${\rm{\Delta }}{\rho }_{N}={\rho }_{N}-{\rho }_{N-1}$, and *h*_*i*_ is the thickness of each layer. The effective Doppler shift due to the background current field is considered by the *Fr* analysis, in other words, the flow is steady (*c*_*i*_ = 0) and the *Fr* becomes unity (*F*_*i*_ = 1) when the intrinsic phase speed and the opposite current speed match (zero apparent speed)^2,3^. Both the surface *Fr* (*F*_*i*_) and the composite *Fr* for the water column (*G*) obtained from the model are of primary interest here to test consistency with the estimated timing of NIW generation prior to the time of each SAR observation.

Then, the distance *D*(*θ*, *t*) over which the NIWs propagate following generated (*t* = 0) in the direction *θ* was calculated by time-integrating the apparent free wave speed, which is the sum of the intrinsic speed and the directional component of the advective speed as a function of direction and time, as shown below:

3 $$D(\theta ,t)={\int }_{0}^{t}{c}_{1}\,dt={\int }_{0}^{t}(g^{\prime} {h}_{1}+{u}_{1}\,\cos \,\theta +{v}_{1}\,\sin \,\theta )dt\,$$

Here, *θ*_0_ is set to the flow direction at the time when the free propagation of NIWs was favorably initiated (*t* = 0) to best fit the SAR observations. Surface-layer current *u*_1_, *v*_1_ and density stratification such as reduced gravity ($g^{\prime}$) to estimate the apparent free wave speed at the surface layer (*c*_1_) were used to calculate the distance.

### Surface patterns

**Figure 3:**
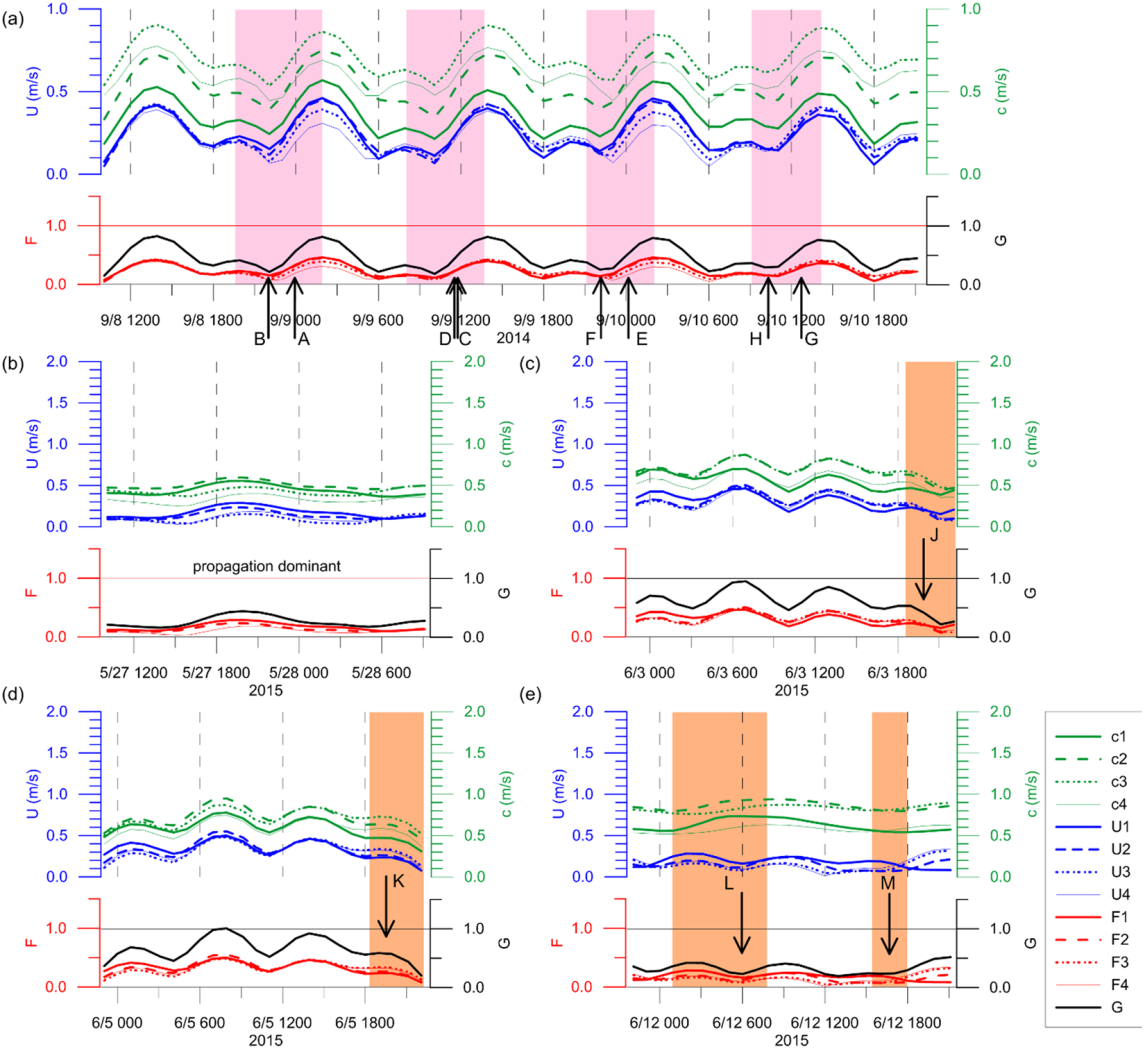
Time series of the advective flow speeds (U), apparent free wave speeds (**c**), and corresponding Froude numbers (F and G). Time series of the advective flow speeds (blue), apparent free wave speeds (green), and corresponding Froude numbers (red) and composite Froude numbers (black) (**a**) from 10:00 UTC September 8 to 22:00 UTC September 10, 2014, (**b**) from 10:00:00 UTC May 27 to 10:00:00 UTC May 28, 2015, (**c**) from 22:00:00 UTC June 2 to 22:00:00 UTC June 3, 2015, (**d**) from 22:00:00 UTC June 4 to 22:00:00 UTC June 5, 2015, and (**e**) from 22:00:00 UTC June 11 to 22:00:00 UTC June 12, 2015. Vertical arrow with labels corresponding to the NIW fronts shown in Fig. 1f indicates when the free wave propagation was favorably initiated or NIW front is presumably formed to fit the SAR observations, which generally correspond to periods of small Froude number (G; thick solid black). Significant decreases in the Froude number to yield subcritical conditions are remarked with pink and orange shades.

**Figure 4:**
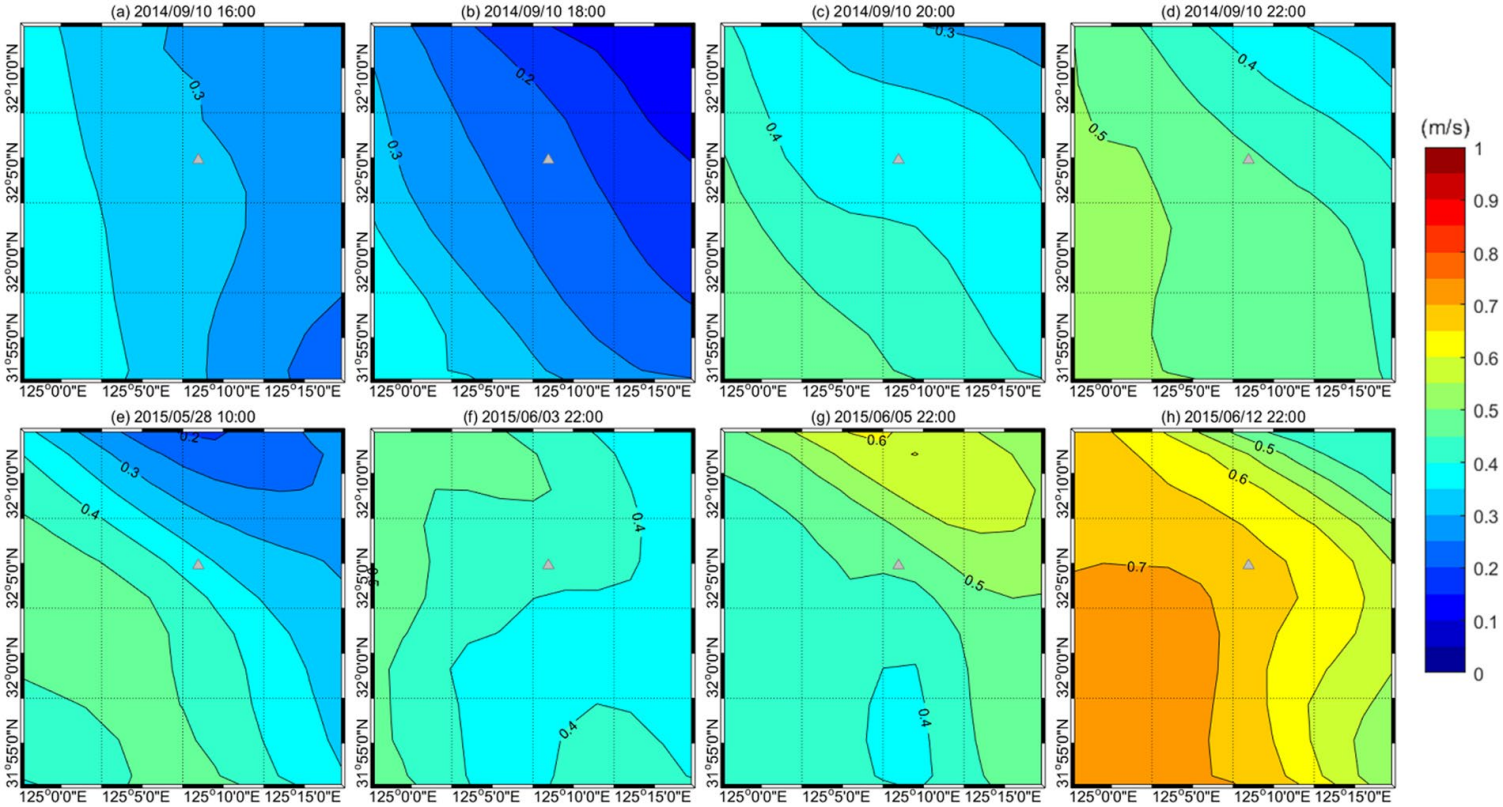
Horizontal map of apparent free wave speed in the vicinity of the IORS. Apparent free wave speed in m s^−1^ (*c*, color bar in the right) incorporating an effective Doppler shift due to the advective flow (speed: *U*) at the surface (*c*_1_) for cases of (**a**) 16:00, (**b**) 18:00, (**c**) 20:00, and (**d**) 22:00 in September 10, 2014, (**e**) 10:00 May 28, (**f**) 22:00 June 3, (**g**) 22:00 June 5, and (**h**) 22:00 June 12, 2015. The sill location is denoted by grey triangle in each plot.

The resulting *Fr* time series indicates that the surface and interior flows across the sill were subcritical (*F*_*i*_ < 1 and *G* < 1) one day prior to the acquisition time of SAR image #2 (May 28, 2015, Fig. 3b) but close to unity (*F*_*i*_ ∼ 1 or *G* ∼ 1) a few to several hours prior to the other SAR image times (#1, #3, #4, and #5 on September 10, 2014, June 3, 2015, June 5, 2015, and June 12, 2015, respectively; Fig. 3a,c,d and e). Thus, the flow regime was “propagation dominant” on May 28, 2015 due to weak advective flow (corresponding to neap tides), supporting no local generation of new NIWs prior to the time of SAR image #2, and the NIW fronts observed in the SAR image #2 (feature ‘I’ in Fig. 1) are not interpreted as the ones locally formed at the sill but rather propagated from a remote source, i.e., somewhere east of the sill when considering the curvature. In contrast, the other NIW fronts observed in the SAR images #1, #3, #4, and #5 (features from ‘A’ to ‘H’ and from ‘J’ to ‘M’ in Fig. 1) are reasonably assumed to be locally generated at the sill when the latest flow regimes become near-critical (*F*_*i*_ ∼ 1 and/or *G* ∼ 1) due to changes in advective flows and density stratification (Fig. 3a,c,d and e). The near-critical flow regime, however, does not mean there is no NIWs of remote source. There are other NIW features than the ones labeled in the SAR images (Fig. 1) which may or may not be generated at remote locations. Our focus here is on the leading wave fronts labeled from ‘A’ to ‘H’ where all but ‘I’ are interpreted as the ones locally generated at the sill. After the *Fr* becomes small enough (subcritical) to allow the NIWs propagate (e.g., vertical arrows in Fig. 3a,c,d and e), the NIW fronts formed locally can be seen in the SAR images, experiencing severe refraction as apparent free wave speed (*c*_*i*_) significantly varies in space and time (Fig. 4).

**Figure 5:**
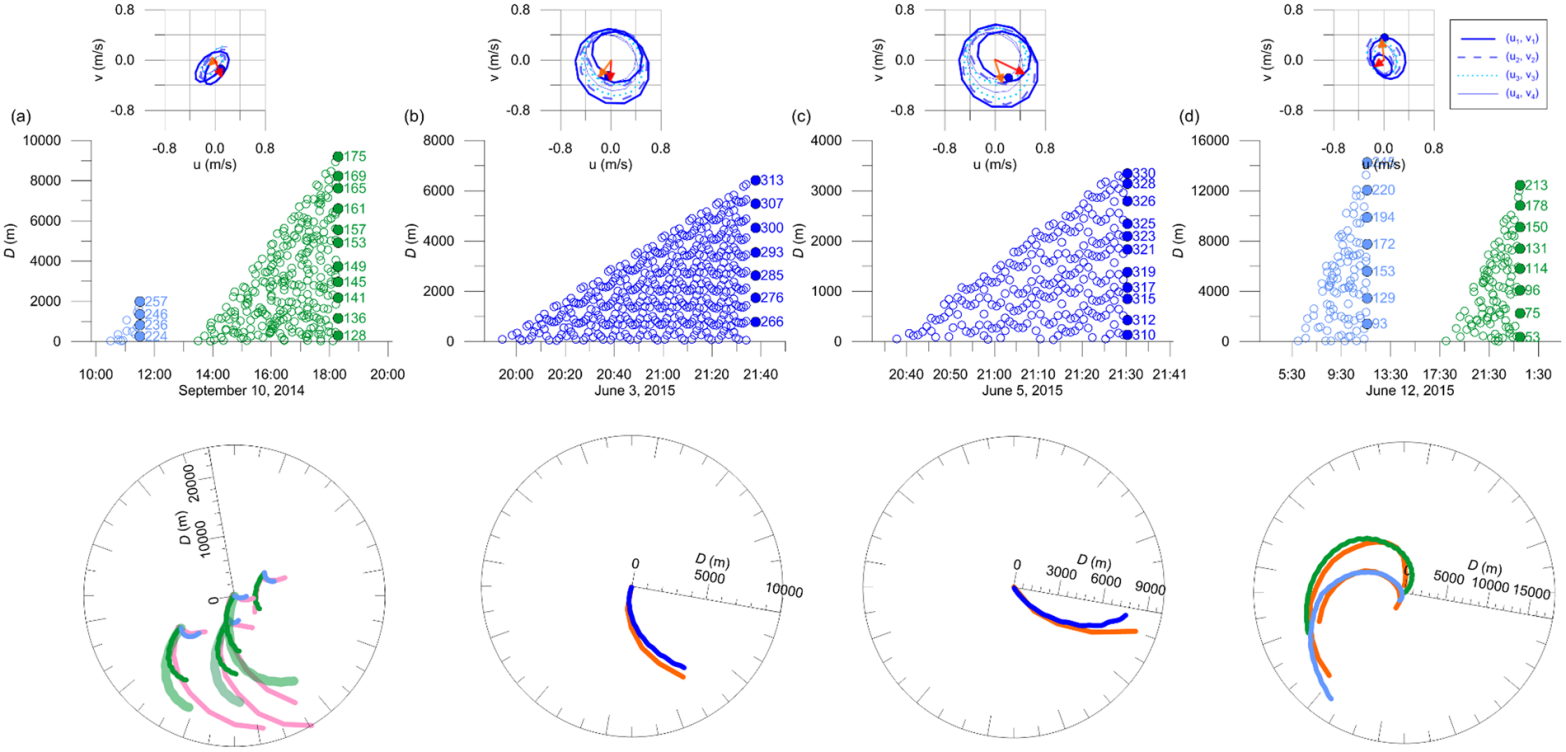
Time evolution of observed and modeled fronts of internal waves in the vicinity of the IORS. Time series of directional (subsampled) measures of distance *D* in meters (y-axis), from the topographic sill (origin) to the internal wave fronts as functions of time *t* (x-axis) and direction *θ* in degrees rotated counterclockwise from the east (numbers labeled in the right of filled circle symbols in the upper plots) for cases of (**a**) September 10, 2014, (**b**) June 3, (**c**) June 5, and (**d**) June 12, 2015. In the lower panel, surface expressions of leading internal wave fronts ((**a**) from ‘A’ to ‘H’ in September 8–10, 2014; (**b**) ‘J’ in June 3, 2015; (**c**) ‘K’ in June 5, 2015; (**d**) ‘L’ and ‘M’ in June 12, 2015) observed by the SAR images (pink for 2014 and orange for 2015) and estimated (cyan and green for successive but distinct waves; (**a**) and (**e**), and blue for a single wave; (**b**) and (**c**)) are compared in the polar coordinate. In (**a**) and (**d**), multiple local origins (arbitrarily shifted) are used for better comparisons. Thick green shaded lines in (**a**) denote the cases in which free wave speeds *c*_*i*_ are doubled to show the sensitivity of the estimation to density stratification. In each panel, hodographs of horizontal current vectors at the IORS reproduced by the ROMS are shown above. They correspond to absolute horizontal (tidal plus subtidal) current vectors (*u*_*i*_, *v*_*i*_) at the four layers (centered at 5, 15, 25, and 35 m) of the station (**a**) from 22:00 UTC September 9 to 22:00 UTC September 10, 2014, (**b**) from 22:00:00 UTC June 2 to 22:00:00 UTC June 3, 2015, (**c**) from 22:00:00 UTC June 4 to 22:00:00 UTC June 5, 2015, and (**d**) from 22:00:00 UTC June 11 to 22:00:00 UTC June 12, 2015. Here, the surface layer current vectors (*u*_1_, *v*_1_) at the starting time, time of wave generation, and the SAR image acquisition time are remarked with blue circles, red arrows, and orange arrows, respectively.

Patterns of the NIW fronts are figured using the estimated directional distance *D* and compared to the SAR observations. Short arc-like patterns of NIW fronts in the SAR images #3 and #4 (features ‘J’ and ‘K’ in Fig. 1) are reproduced with NIW generations 1.72 and 1.78 hours prior to the SAR acquisition times respectively (Table 1). Note that the initial (*t* = 0) directions of NIW propagation from the sill (origin) are *θ*_0_ = 273.9 for image #3 and *θ*_0_ = 327.2 for image #4, following the tidal flow (red vectors in Fig. 5b and c). The propagation of NIW fronts from the initial times to the SAR acquisition times (Table 1) rotating clockwise (from red to orange vectors in Fig. 5b and c) around and away from the sill yields the short arc-like patterns similar to the SAR observations (blue vs orange in Fig. 5b and c). This is consistent with results of the *Fr* number analysis, as *Fr* is close to unity (near-critical flow, e.g., *F*_*i*_ ∼ 1 and/or *G* ∼ 1) with advection speed (*U*_*i*_) comparable to the intrinsic free wave speed (*c*_*i*_ − *U*_*i*_) and decreases to yield subcritical condition (*F*_*i*_ < 1 and *G* < 1) during the time of decelerating tidal flow (shaded in Fig. 3c and d).

Long and spiral patterns originating from the sill are found in SAR image #5. The features ‘L’ and ‘M’ appear to be linked to NIW generation 15.7 and 4.9 hours prior to the SAR image acquisition times, respectively (Table 1). Similarly, the flow was subcritical with decelerating advective flow and *Fr* is sufficiently less than unity (arrows in Fig. 3e). Interestingly, the Froude number at the surface layer (*F*_*i*_) approached local minimum first at around 06:00 (‘L’) and once more at around 17:00 (‘M’) on June 12, 2015, re-initiating the NIW generation with an approximate semidiurnal period (between the first and second arrows in Fig. 3e). The resultant distance *D* of the NIW fronts, with *θ*_0_ = 245.3 and *θ*_0_ = 212.6 before and after the re-initiation, respectively (the latter is shown with a red arrow in Fig. 5d), yields two long spiral patterns where the one formed before the re-initiation was arbitrarily shifted in the southwest direction for better comparison to the SAR observations (cyan vs orange in Fig. 5d). Thus, the two long spiral patterns in SAR image #5 are successfully reproduced using the estimated *D* with the re-initiation due to changes in advective flow speed and stratification or *Fr* conditions (Fig. 5d).

Here, our focus is on the leading wave front yet a number of parallel waves in a packet are seen from multiple wave fronts. It is typical that such a nonlinear internal soliton disintegrates into rank-ordered multiple solitary waves (fission process) while propagating and evolving^18,27^. Changing stratification and bathymetry following the propagating and evolving NIWs account for the disintegration and fission processes^28–30^.

More similar to what is found in SAR images #3 and #4 (features ‘J’ and ‘K’ in Fig. 1) than those in the SAR image #5 (‘L’ and ‘M’), multiple short arc-like or wedge patterns were observed in the SAR image #1 (features ‘A’ to ‘H’ in Fig. 1). Four pairs of wedge patterns (‘A’ and ‘B’, ‘C’ and ‘D’, ‘E’ and ‘F’, and ‘G’ and ‘H’) are generated at intervals of quasi-semidiurnal periods with *θ*_0_ ranging from 251.8 to 347.2 (Fig. 5a), where the latest one (‘H’) is shown in Fig. 5a (red arrow). Following the semidiurnal tidal cycle and diurnal inequality, the *Fr* reaches the minimum twice a day with a subcritical flow condition when the NIWs re-initiate and start to propagate (arrows in Fig. 5a). Thus, diurnal inequality in tidal current speed and consequent changes in *Fr* account for the wedge features found in the SAR image #1 (Fig. 1a,f, or pink in Fig. 5a), reproducing shorter features ‘B’, ‘D’, ‘F’, and ‘H’ (cyan in Fig. 5a) and longer features ‘A’, ‘C’, ‘E’, and ‘G’ (green in Fig. 5a). The longer features of the wedge patterns ‘C’, ‘E’, and ‘G’ are more comparable to the SAR observation when the intrinsic free wave speed or reduced gravity arbitrarily doubled (thick green shaded lines in Fig. 5a), implying that better agreements would be possible with advancements to future modeling skills. The time intervals between ‘A’-‘B’ and ‘C’-‘D’, between ‘C’-‘D’ and ‘E’-‘F’, and between ‘E’-‘F’ and ‘G’-‘H’ do not exactly match but close to semidiurnal lunar tidal cycle following the dominant tidal forcing. It is because the flow regime is not only function of the tidal current but also free wave speed (*c*_*i*_) imposed by stratification, non-tidal component of *U*_*i*_, apparent free wave speed (*c*_*i*_ − *U*_*i*_), and consequently *Fr* condition. Note that the stratification may also vary significantly particularly when internal tides become significant (occasionally true but not always).

## Conclusions

In summary, arc-like, wedge, and spiral patterns were found in the surface expression of oceanic NIWs in the vicinity of small topographic sills, IEODO, in the northern ECS in September 2014 and May to June 2015. Series of analyses of satellite SAR images taken in the region along with background conditions on horizontal flow and density stratification reproduced by the ROMS reveal that the NIWs were locally generated when the flow approached near-critical Froude number conditions (*Fr* is close to unity) and started to propagate when the flow became subcritical (*Fr* < 1), and that the NIW energies radiated from the sill in all directions but with a significant time lag primarily dependent on the conditions of tidal flow and stratification. The position of the NIW fronts as a function of direction and time is simply modeled and compared with the SAR observations, as determined by the time-varying advective flow speed (mostly semidiurnal tidal currents) and density stratification (intrinsic free wave speed). The modeled NIW fronts are surprisingly similar to the observations, providing all the short arc-like, wedge, and spiral patterns. How the NIW fronts generated at and radiated from the sill will ultimately dissipate in the ECS remains an open question. The abundance of topographic features like sills, islands, and shelf breaks in the ECS support more complex NIW processes including vigorous wave-wave interaction. These mechanisms, in addition to the one presented here, will be realized for any broad shelf having a shallow sill or island with strong time-varying and/or rotary (such as tidal) flows. The significance of this finding lies in the unprecedented observations of a new type (wedge and spiral patterns) of NIW fronts radiating from a shallow sill topography on the broad shelf, which has important implications for processes transporting and redistributing heat, momentum, energy, and materials in the global coastal ocean as well as the ECS.

## Methods

The IEODO, also known as Socotra rock, is a submerged rock 4.6 m below mean sea level located in the northern ECS where an ocean research station, the IORS was built in 2003. Long and continuous time-series of oceanographic and meteorological parameters (wind speed and wave height) have been collected at the station since then. Here, we mainly used the time-series data collected in 2014 and 2015. Other data, such as vertical profiles of water temperature and salinity intermittently collected (with use of SBE Conductivity-Temperature-Depth; CTD) flow velocities at multiple levels (with use of the bottom-mounted TRDI acoustic Doppler current profiler (ADCP) near the station; no data are available for the periods of 2014 and 2015 but 2008), sea surface temperature remotely sensed from the station (with use of thermal infrared radiometer), were used to verify general characteristics of the vertical density stratification, free wave speed, and characteristics of tidal currents as well as the internal wave fronts.

Five SAR images taken in the vicinity of IORS in September 2014 and May to June 2015 were used to find frontal features associated with NIWs. All SAR images were firstly geolocated using the ancillary information provided with the SAR data and a small translation was applied to coincide the IORS signature (bright point) between SAR images. The NIW fronts were delineated along the maximum gradient of leading NIW seen in the SAR images. Specification of the SAR images such as frequency band, orbit direction, polarization, incident angle, and sea surface wind and wave conditions are listed in Table 2. Time series data collected at the IORS were used to extract the sea surface wind and wave conditions at the SAR image times.

A research cruise, named the shallow-water acoustic variability experiment (SAVEX-15) was conducted using the Korean R/V Onnuri during 14–28 May 2015 in the northern ECS, ~50 km east of the IORS. Under the goal to obtain simultaneous oceanographic and underwater acoustic data appropriate for investigating the coupling of oceanography and underwater communications in the region^31^, the vessel mounted 150 kHz ADCP and both stationary and underway CTDs (with 26 and 1,062 casts respectively) were used to measure temporal and spatial variations of flow velocities at multiple depths (water depth is about 100 m) and density stratification, respectively. An abundance of isotherm or isopycnal undulations at semidiurnal and higher frequencies was confirmed from the CTD data. Horizontal currents at the upper (25 m) and lower (65 m) depths, which are dominated by semidiurnal tidal currents, observed from the ADCP during the SAVEX-15, were used to verify the reproduced currents (Fig. 2).

Regional scale circulations with background conditions (density stratification and horizontal currents) were simulated with use of the ROMS. The modeled area covers the Yellow Sea and the ECS (117.5–130.3°E, 24.8–41°N). Ocean circulation in the Yellow Sea and ECS was spun-up using open boundary conditions imposed with the data from the Northwest Pacific model^32^. The horizontal grid size is ~3 km and number of vertical levels are 20. The horizontal viscosity was set to 300 m^2^ s^−1^. Climatological variations of discharges from the Changjiang River and the Huanghe River were included as freshwater sources. Tidal effects were included in the numerical simulations along the open boundary using eight major tidal components derived from TPXO6^33^. Meteorological data predicted by the Korea Meteorological Administration were used to calculate surface wind stress and surface heat flux. The calculated tides were verified through comparisons with observations from tidal stations located at the IORS from April 2015 to June 2015 (Fig. 2e). Further details of the ROMS follow previous literatures^27^.
